# Cancer immunogenic cell death via pyroptosis with CXCR4-targeted nanotoxins in hepatocellular carcinoma

**DOI:** 10.3389/fbioe.2024.1433126

**Published:** 2024-11-04

**Authors:** Yingbin Huang, Yihu Li, Rui He, Shuyi Dong, Zheng Zhao, Xingyuan Jiao

**Affiliations:** ^1^ Organ Transplantation Center, The First Affiliated Hospital, Sun Yat-Sen University, Guangzhou, China; ^2^ Department of Hepatobiliary Surgery, The Second Affiliated Hospital, Guangzhou Medical University, Guangzhou, China; ^3^ State Key Laboratory of Oncology in South China, Guangdong Key Laboratory of Nasopharyngeal Carcinoma Diagnosis and Therapy, Guangdong Provincial Clinical Research Center for Cancer, Sun Yat-Sen University Cancer Center, Guangzhou, China

**Keywords:** targeted therapies, CXCR4, pyroptosis, immune microenvironment, hepatocellular carcinoma

## Abstract

**Introduction:**

Cytotoxic agents have shown limited benefits in hepatocellular carcinoma (HCC), mediated in part by the lack of targeting. As cell-penetrating peptides (CPPs) are capable of delivering various biologically active molecules into cells, including protein, peptides, small chemo-drugs, and nucleic acid with or without targeting, we developed T22-PE24, a CXCR4-targeted self-assembling cytotoxic nanotoxin, to effectively induce HCC pyroptosis.

**Methods:**

T22 incorporating *enhanced green fluorescent protein (*EGFP) or PE24 was purified from DE3 bacterial cells and characterized using transmission electron microscopy, the Zetasizer Nano^®^, and SEC-HPLC. The internalization effect of T22-EGFP was detected by flow cytometry system (FCS) in CXCR4^+^/LM3(^CXCR4−^) HCC cells. The CCK8, lactate dehydrogenase (LDH) release, Western blot, and nude mice HCC models were used to estimate the cell viability of T22-PE24. The complete-immunity HCC tumor-bearing mice model was used to assess the immune response of T22-PE24.

**Results:**

The round shape under transmission electron microscopy, 49.4 nm hydrodynamic diameter, and −33.33 mV zeta potential indicated that T22-PE24 self-assembled into nanoparticles. T22 incorporating EGFP selectively internalized in CXCR4^+^ HCC cells and showed no accumulation in CXCR4-knockout HCC cells. The T22-PE24 nanotoxin induced HCC pyroptosis via the caspase-3/GSDME signaling pathway and suppressed tumor growth in the absence of histological alterations in normal organs. Using the complete-immunity HCC tumor-bearing mice model, we found that T22-PE24 nanotoxin effectively induces the global reprogramming of cell components of the immune tumor microenvironment, leading to enhanced antitumor effects compared to those observed in immunodeficient mice.

**Conclusion:**

Our findings demonstrate the activation of the innate immune response in HCC by inducing pyroptosis with T22-PE24 nanotoxin treatment and support an implementation of this strategy for HCC treatment.

## Introduction

Hepatocellular carcinoma (HCC) is among the most lethal malignancies worldwide, with a high mortality rate and dismal prognosis (Chon et al., 2021; Siegel et al., 2021). Current therapeutic options are limited, and most chemotherapy selections are cytotoxic agents, such as sorafenib (Anwanwan et al., 2020; Vogel et al., 2022). The potential of cytotoxic agent-based therapies in HCC therapy has been known for years, and various promising treatments have gained approval from the US Food and Drug Administration (El-Serag et al., 2008; Salem et al., 2022). The bacterial toxins *Pseudomonas* exotoxin (PE) and *diphtheria* toxin A fragment and their derivatives have been proven to possess adenosine diphosphate (ADP)-ribosylation activity (Falgas et al., 2020a; Nunez et al., 2023; Rioja-Blanco et al., 2022a). In the cytosol, the enzymatically active C-terminal fragment of these toxins transfers ADP-ribose from nicotinamide adenine dinucleotide to eukaryotic elongation factor 2, inhibiting protein biosynthesis and ultimately leading to cell death (Gebhardt et al., 2014; Moradian and Rahbarizadeh, 2021). PE24 is a truncated *Pseudomonas* exotoxin lacking the immunized catalytic domain (Lee et al., 2019; Morgan et al., 2023). Unfortunately, many toxins, including PE24, exhibit a lack of targeting and cause serious side effects, but those effects can be eradicated when coupled with a cell-penetrating peptide (CPP) (Liu et al., 2016; Takeuchi et al., 2006; Yu et al., 2021b). These CPPs should selectively bind to and kill cancer cells while sparing normal cells. Different strategies have been actively developed to improve cytotoxic agent therapy in HCC, with a major focus on combining CPPs with other existing therapies (Jin et al., 2018; Wang et al., 2018). Such combinations have been shown to increase antitumor efficacy with fewer side effects in animal models and clinical trials (Gusarova et al., 2007; Zhao et al., 2018). However, responses are seen only in a limited fraction of patients, mainly attributed to therapy resistance and the lack of activated immune cells. Thus, new combinatorial strategies are still urgently needed.

A high expression of CXCR4, the receptor of CXCL12, has been identified to be associated with tumorigenesis, progression, the risk of metastasis, and the overall median survival in HCC, which is a validated selective target in HCC (Yang et al., 2019; Yang et al., 2020). To improve the targeting of HCC cells by cytotoxic agents, we attempted to fuse PE24 with CXCR4-targeting peptide T22 (RRWCYRKCYKGYCYRKCR). T22-PE24, recombinantly produced in *Escherichia coli*, self-assembles into multimeric nanoparticles. The T22-PE24 nanotoxin has already proved to be effective in targeting CXCR4^+^ cells in head and neck squamous cell carcinoma (HNSCC) (Rioja-Blanco et al., 2022a), diffuse large B-cell lymphoma cells (DLBCL) (Falgas et al., 2020a), colorectal cancer (CRC) (Sala et al., 2022), endometrial cancer (EC) (Medina-Gutierrez et al., 2022), and acute myelocytic leukemia (AML) (Nunez et al., 2023). Remarkably, no CPP-cytotoxic-loaded nanotoxin has been reported to selectively target HCC cells, highlighting the relevance of this study.

Tumors use various strategies to avoid or limit the cell death pathway (Cerella et al., 2014). Much evidence shows that pyroptosis can activate the inflammasome and release pro-inflammatory cytokines, contributing to the remodeling of the tumor immune microenvironment (Shao, 2021; Yu et al., 2021a). The occurrence of pyroptosis in a few tumor cells can trigger potent innate immune responses, ultimately resulting in effective tumor regression (Wang et al., 2020). Developing new and specific therapeutic approaches to induce pyroptosis may benefit HCC treatment.

In this study, we aimed to investigate the effects of T22-PE24 on HCC using both cell lines and mouse models. CXCR4 was highly expressed in HCC cells, and T22 was especially targeted in CXCR4^+^ HCC cells. The T22-PE24 treatment of HCC is mediated via pyroptosis induction and cell proliferation arrest. In addition, in a complete-immunity HCC tumor-bearing mice model, T22-PE24 treatment significantly accumulated in a majority of activated immune cells and acute HCC cells. Our results suggest that T22-PE24 could prove to be an invaluable tool for evaluating the therapeutic potential of CPP and cytotoxic agents.

## Materials and methods

### T22-PE24 purification and characterization

T22-EGFP (enhanced green fluorescent protein; amino acid sequence: RRWCYRKCYKGYCYRKCRGGSSRSSMSKGEELFTGVVPILVELDGDVNGHKFSVSGEGEGDATYGKLTLKFICTTGKLPVPWPTLVTTFSYGVQCFSRYPDHMKQHDFFKSAMPEGYVQERTIFYKDDGNYKSRAEVKFEGDTLVNRIELKGIDFKEDGNILGHKMEYNYNSHNVYIMADKQKNGIKVNFKIRHNIEDGSVQLADHYQQNTPIGDGPVLLPDNHYLSTQSALSKDPNEKRDHMILLEFVTAAGITHGMDELYK) and T22-PE24 (amino acid sequence: RRWCYRKCYKGYCYRKCRGGSSRSSRHRQPRGWEQLGGSPTGAEFLGDGGDVSFSTRGTQNWTVERLLQAHAQLEERGYVFVGYHGTFLEAAQSIVFGGVAARSQDLAAIWAGFYIAGDPALAYGYAQDQEPDAAGRIRNGALLRVYVPASSLPGFYRTSLTLAAPEAAGEVERLIGHPLPLALDAITGPEEEGGRLETILGWPLAERTVVIPSAIPTDPRNVGGDLDPSSIPDKEQAISALPDYASQPGKPPREDLK) with 6 × His-tag coding sequences were introduced in the plasmid pET22b by Gencefe (Zhejiang, China). Plasmids were transformed into DE3 bacterial cells, and recombinant gene expression was induced overnight at 20°C by 0.1 mM isopropyl-β-D-thigalactopyronaside (IPTG). Cells were harvested and resuspended in Tris buffer (Tris 20 mM, NaCl 500 mM, pH 8.0, imidazole 10 mM) in the presence of a cocktail protease inhibitor (TargetMol, #C0001). Cells were then disrupted by high pressure and centrifuged at 30,000 g at 4°C for 60 min. The supernatant was incubated for 2 h at 4°C with pre-equilibrated Ni Sepharose excel (Cytiva, #17371202) and then passed through a gravity column (Cytiva, #17043501) for gravity flow purification. The column was washed using resuspension buffer, and the protein was eluted using the same buffer with 500 mM imidazole. Proteins were finally aliquoted in small 50 μL samples in phosphate-buffered saline (PBS) and stored at −80°C after 0.22 μm pore membrane filtration.

The size and surface morphology of nanoparticles were studied using transmission electron microscopy (TEM). The size and Z-potential were determined using the Zetasizer Nano^®^ (PSA; Zetasizer Nano ZS, Malvern Instruments Limited, Malvern, Worcestershire, United Kingdom). Size exclusion-high-performance liquid chromatography (SEC-HPLC) was performed on an Agilent 1260 Infinity Quaternary LC System with a diode array detector (DAD). The isocratic separation for proteins was achieved on an Agilent AdvanceBio SEC 300 Å (7.8 mm × 300 mm, 2.7 μm) column using a mobile phase of PBS at a flow rate of 0.5 mL/min at ambient temperature. All the proteins were detected using a UV detector at 280 nm. The Agilent OpenLAB ChemStation Edition software was used for data analysis. The mass spectrometry was analyzed by WininnovateBio (Shenzhen, China). The endotoxin content of the target proteins was determined using End-point Chromogenic Tachypleus Amebocyte Lysate (BIOENDO, Xiamen, China) according to the manufacturer’s protocol. The endotoxin content was determined using a standard curve: Y = a × X + b, r > 0.96 (Y = OD405 nm value, X = endotoxin content).

### Cell lines, cell culture, transfection, and lentiviral transduction

Human HCC cell lines HepG2, LM3, Hep3B, Huh7, LM6, SK-HEP-1, and MHCC97H, human normal liver cell line LO2, and mouse HCC cell lines Hepa1-6 were maintained in Dulbecco’s Modified Eagle’s medium (DMEM) (Sigma-Aldrich, #D6429-500 mL) supplemented with 10% fetal bovine serum (FBS) (VISTECH, #SE100-B) and 100 U/mL of penicillin/streptomycin (ThermoFisher, #15140122). All cells were cultured in a humidified incubator with 5% CO_2_ at 37°C.

Small interfering RNA (siRNA) for GSDMA, GSDMB, GSDMC, GSDMD, and GSMDE were designed and synthesized by Guangzhou Ruibo Biotechnology Co., Ltd. Their comparison is denoted as negative control small interfering RNA (siNC). The sequences were as follows:

siGSDMA: CAA​AGA​CGG​UGA​AGG​UGA​A

siGSDMB: GAC​UCA​ACG​GGA​GAG​UUG​A

siGSDMC: GAA​GGA​UUC​UCG​UUC​AUC​A

siGSDMD: GGA​ACT​CGC​TAT​CCC​TGT​T

siGSDME: GAA​UGA​CUC​UGA​UAA​GUU​A

siNC: unrevealed.

For one well of a 24-well plate, 3 µL of 20 µM siRNA was mixed with 50 µL of Opti-MEM in one tube. In another tube, 3 µL of lipofectamine RNAiMAX (Invitrogen, #1377815) were mixed with 50 µL of Opti-MEM and incubated for 10 min at room temperature. The solutions were combined and gently mixed by inversion and incubated for another 15 min at room temperature. The 100 µL of siRNA-RNAiMAX were added into cultured cells (40%–50% confluent). The efficiency of transfection was determined by Western blot after 48 h.

Lentivirus from Fulen Gen (#HCP218803-LvSG06, unrevealed sgRNA sequence) was used to knock out CXCR4. In brief, LM3 or Hep3B cells were seeded onto a 6-well plate for 12 h and then treated with either vector lentivirus or sgCXCR4 lentivirus for 6 h. The cells were continuously cultured in complete medium for 48 h, and then the Beckman Coulter MoFlo Astrios Cell Sorter was used to select cells with CXCR4-negative expression. These selected cells were cultured in complete medium supplemented with 1 μg/mL of puromycin.

### Real-time PCR

Total RNA was extracted by TRIzol reagent (ThermoFisher, #15596018). A 2 μg sample of total RNA was converted into cDNA using the HiScript III All-in-one RT SuperMix Perfect for qPCR (Vazyme, #R333-01). The cDNA was amplified using the following specific primers:

CXCR4 forward primer: AGC​ATG​ACG​GAC​AAG​TAC​C.

CXCR4 reverse primer: GAT​GAT​ATG​GAC​AGC​CTT​ACA​C

β-actin forward primer: GAT​CAA​GAT​CAT​TGC​TCT​CCT​G

β-actin reverse primer: AGG​GTG​TAA​AAC​GCA​GCT​CA.

All primers were synthesized by RuiBiotech (RuiBiotech, Beijing, China). The 2 × ChamQ SYBR qPCR Master Mix (Vazyme, #Q311-03) was used for PCR reactions, which were run on a Bio-Rad CFX96 PCR system. The PCR amplifications were performed at 95°C for 15 s, followed by 40 cycles at 95°C for 10 s, 60°C for 30 s, and 95°C for 15 s. The melting curves were performed to validate the utility and specificity of the PCR product. The ratio of the mRNA expression relative to the control was evaluated using the comparative CT method.

### Live cell imaging and cell viability assays

The toxicity studies of T22-PE24 were determined using the cell counting kit-8 (CCK8; Dojindo, #CK04) experiment. In brief, 0.5 × 10^4^ cells were seeded in a 96-well plate and cultured overnight. Cells were incubated for 24 h with either different concentrations of T22-PE24 or phosphate-buffered saline (PBS). Morphological changes, if any, were visualized by IncuCyte (Essen Bio Science Inc.). The cells were then incubated with the CCK8 reagent for 3 h before determining the absorbance at 450 nm using a multi-well spectrophotometer (Bio-Tek EPOCH). The percentage of cell viability was calculated for each concentration of T22-PE24 by plotting the cell viability against the logarithm of T22-PE24 concentration and determining the IC_50_ (the concentration at which 50% of cells are inhibited) value using non-linear regression analysis by GraphPad Prism.

### LDH release assay

LDH release upon T22-PE24 exposure was studied using the LDH Cytotoxicity Assay Kit (Beyotime, #C0017). A total of 0.5 × 10^4^ cells were seeded in a 96-well plate and cultured overnight. The cells were incubated with 1 μM T22-PE24 for 24 h followed by LDH detection.

### Flow cytometry

For the T22-EGFP-penetrating experiment, cells were cultured with different concentrations of T22-EGFP for 4 h in fresh medium. After digestion by trypsin, the fluorescence of EGFP in cells was detected by CytoFLEX LX (Beckman Coulter).

To evaluate the effect of T22-PE24 on HCC cells, 2 × 10^5^ cells were seeded in a 24-well plate and cultured overnight. The cells were then treated with either PBS or T22-PE24 for 24 h. They were collected in a 1.5 mL EP tube and stained with Annexin V-FITC/propidium iodide (PI) (KeyGEN, #KGA108) for 20 min. The fluorescence was detected by a CytoFLEX LX (Beckman Coulter).

### Western blotting

Western blotting was performed to detect protein expression levels of caspase-3, cleaved caspase-3, PARP1, cleaved PARP1, GSDME, NT GSDME, and GAPDH. After treatment with nanotoxin or caspase inhibitor, the cells were harvested and washed using cold PBS. After incubating in an ice-cold RIPA lysis buffer (Beyotime, #P0013B), the cell lysates were centrifuged (8,000 g, 10 min) and harvested. The protein concentration was determined using a BCA Protein Assay Kit (ThermoFisher, #23225). Protein samples (30–50 μg) were separated by SDS–polyacrylamide gel electrophoresis and transferred to polyvinylidene difluoride membranes. The membranes were incubated in 5% non-fat milk at room temperature (RT) for 60 min and then incubated in primary antibody caspase-3 (Abcam, #ab32351), cleaved caspase-3 (Abcam, #ab32042), cleaved PARP (Cell signaling technology, #9541S), NT GSDME (Abcam, #ab222407), and GAPDH (Cell signaling technology, #2118S) at a 1:1000 dilution at 4°C overnight. The membranes were then washed with PBST (PBS with 0.1% Tween-20) and incubated with secondary antibodies [anti-mouse IgG HRP-linked antibody (Cell signaling technology, #7076S), anti-rabbit IgG HRP-linked antibody (Cell signaling technology, #7074S)] at 1:5,000 dilution at RT for 60 min. The membranes were visualized by enhanced chemiluminescence (ECL) (Zen BioScience, #17046-500 mL) and imaged with the Tanon 5200 Multi Chemiluminescence Imaging System (Shanghai, China) as recommended by the manufacturer.

### *In vivo* experiments

Female BALB/c nude mice (5–6 weeks) and C57 mice (5–6 weeks) were purchased from Charles River (Zhejiang, China) and kept under a specific pathogen-free (SPF) environment with free access to standard food and water. All animal experiments were approved by the ethics committee of Sun Yat-sen University (approval No. L025503202205014). A total of 5 × 10^5^ Hepa1-6 cells were subcutaneously injected into the back of C57 mice, and 5 × 10^5^ LM3 cells were subcutaneously injected into the back of BALB/c nude mice. The tumor volume was calculated as follows:

$$tumor\;volume=\frac{length\times {width}^{2}}{2}.$$

The day of inoculation was marked as Day 0; nanotoxin treatment studies were used in mice on Day 5.

For the tumor inhibition of nanotoxin T22-PE24, LM3 tumor-bearing BALB/c nude mice were randomly divided into three groups and were intravenously (i.v.) treated with 100 μL physiological saline or 10 mg/kg or 100 mg/kg of T22-PE24. The treatments were repeated i.v. every 2 days. Subcutaneous Hepa1-6 tumor-bearing mice were also randomly divided into two groups and treated with physiological saline or 10 mg/kg of T22-PE24. The treatment was repeated every 2 days. The tumor size and mouse weight were measured every other day. The hematoxylin-eosin (H&E) stain and imaging were used to assess biosecurity in major organs. A paraffin section of tumor tissues was prepared for histological study (Servicebio Technology, Wuhan, China).

### Blood sample study

At the end of the *in vivo* experiment, mice blood samples were collected from eyeballs. The blood samples from LM3 tumor-bearing BALB/c nude mice were centrifuged to get serum. Alanine aminotransferase (ALT), aspartate aminotransferase (AST), creatinine (Cr), and blood urea nitrogen (BUN) were detected by automatic biochemistry analyzer according to the manufacturers’ procedures (Donglin Bio, Guangzhou, China). The peripheral blood mononuclear cells (PBMC) from Hepa1-6 tumor-bearing mice were separated and collected by lymphocyte separation medium (TBD Science, Tianjin, China). Then, the cells were incubated with eFluor™ 660 anti-CD3 antibody (Invitrogen, #50-0032–82) and Alexa Fluor 488 anti-CD8 antibody (Abcam, #ab237364) for 30 min at 4°C. The cells were resuspended in PBS to remove the excess antibodies and analyzed via flow cytometry.

### Histological study

Fresh tumor tissues were fixed with 4% paraformaldehyde, embedded in paraffin, and then sectioned at 4 μm thickness. Slices were subjected to deparaffinization, antigen retrieval, and blocking. For immunohistochemistry (IHC) analysis, the slices were incubated with anti-cleaved caspase-3 antibody (Abcam, #ab32042) or anti-Ki67 antibody (Servicebio Technology, #GB111499) at 4°C overnight, followed by incubation with anti-rabbit IgG secondary antibody and DAB reagent. The slices were mounted using neutral resins and viewed under a microscope (Nikon Eclipse Ni-U).

For immunofluorescence (IF), slices were stained with eFluor™ 660 anti-CD3 antibody (Invitrogen, #50-0032–82) and Alexa Fluor 488 anti-CD8 antibody (Abcam, #ab237364) for 2 h at room temperature and washed with PBS three times. Then, the slices were mounted with a Diamond Antifade Mountant with DAPI medium (ThermoFisher, #P36962) and imaged by a confocal microscope (Zeiss LSM880).

### Statistical analysis

Each experiment was independently repeated at least three times with similar results. Data are presented as the mean ± SD/SEM, and statistical analyses were performed using GraphPad Prism 8 (GraphPad Software, San Diego, California, United States). The T-test or one-way ANOVA was used to determine the significance of pairwise comparisons, and the log-rank test was applied for the comparison of survival curves. *p <* 0.05 was considered significant. **p < 0.01, ***p < 0.001, ****p < 0.0001.

## Results

### Characterization of T22-EGFP and T22-PE24

T22-EGFP and T22-PE24 were purified from DE3 bacterial cells. The Coomassie blue stain, HPLC, and mass spectrometry showed a purity of over 90% (Supplementary Figures S1A–C). TEM showed clear outlines of T22-PE24 with ultrastructural morphometry (round shape and clear size populations) (Supplementary Figure S1D). The mean hydrodynamic diameter of T22-PE24 was 49.4 nm, and the zeta potential was −33.33 mV (Supplementary Figure S1E). The optimal diameter and zeta potential changes confirmed that T22-PE24 self-assembled into nanoparticles, which would ameliorate the half-life in blood circulation and enhance accumulation in tumor tissues. In our previous study, we found T22-PE24 was stable in stored buffer with or without BSA at −80°C, 4°C, or 37°C for 30 days (Zhao et al., 2023). The aforementioned characteristics indicate that T22 may be an ideal candidate for the sustained delivery of PE24, specifically targeting CXCR4.

### T22 selectively targets HCC cells expressing CXCR4

**FIGURE 1:**
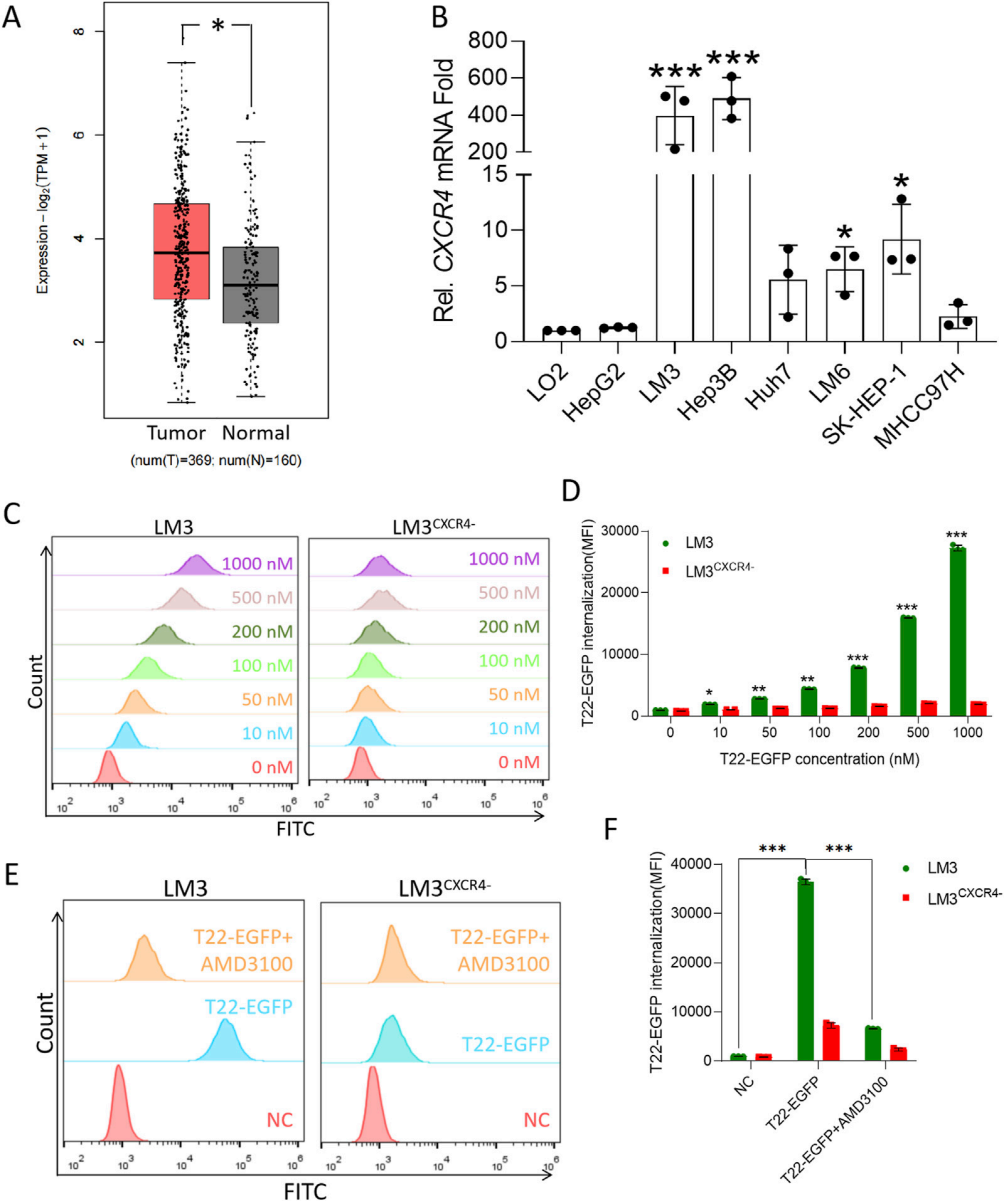
High expression of CXCR4 in HCC cells and T22 targeting HCC. **(A)** CXCR4 expression in HCC cells and the normal tissue analyzed from TCGA. **(B)** CXCR4 expression in HCC cell lines determined by RT-PCR. **(C)** Cellular uptake in LM3 and LM3^CXCR4−^ cells after treatment with various concentrations of T22-PE24 for 4 h by flow cytometry. The results are representative of three independent experiments. **(D)** Quantification analysis of T22-PE24 internalization in **(C)**. **(E)** Cellular uptake in LM3 and LM3^CXCR4−^ cells after treatment with T22-PE24 alone or in combination with CXCR4 inhibitor AMD3100. **(F)** Quantification analysis of T22-PE24 internalization in **(E)**. Mean ± SEM of n = 3 repeats. *p < 0.05, **p < 0.01, ***p < 0.001 in a one-way ANOVA, followed by Tukey’s multiple-comparison test.

CXCR4 in HCC tumor tissues compared to normal tissues is shown in Figure 1A. Additionally, RT-PCR analysis of various HCC cell lines revealed significantly elevated CXCR4 expression in LM3, Hep3B LM6, and SK-HEP-1 cells compared to the normal liver cell line LO2 (Figure 1B). After knocking out CXCR4 in LM3 and Hep3B cell lines, Western blot analysis confirmed minimal to no CXCR4 expression in LM3^CXCR4−^ and Hep3B^CXCR4−^ cells (Supplementary Figures S1F, G). To investigate the permeability and targeting effects of the T22 carrier in HCC, T22-EGFP internalization inside the cells was measured by flow cytometry and is represented as mean fluorescence intensity (MFI). We found that T22-EGFP internalized in LM3 in a concentration-dependent manner, whereas the CXCR4-silencing cell lines LM3^CXCR4−^ displayed no significant internalization compared to buffer controls (no statistically significant differences, Figures 1C, D). Moreover, the selective internalization of T22-EGFP was further corroborated by CXCR4 blockage using CXCR4 antagonist AMD3100. LM3 pretreated with AMD3100 and LM3^CXCR4−^ cells did not internalize T22-EGFP compared to LM3 without AMD3100 pretreatment (Figures 1E, F). In the mouse HCC cell line Hepa1-6, the selective internalization of T22-EGFP could also be inhibited by AMD3100 (Supplementary Figure S2A). Thus, we demonstrated that T22-EGFP internalized within HCC cells via targeting CXCR4.

### *In vitro* cytotoxicity, pyroptosis, and mechanism

**FIGURE 2:**
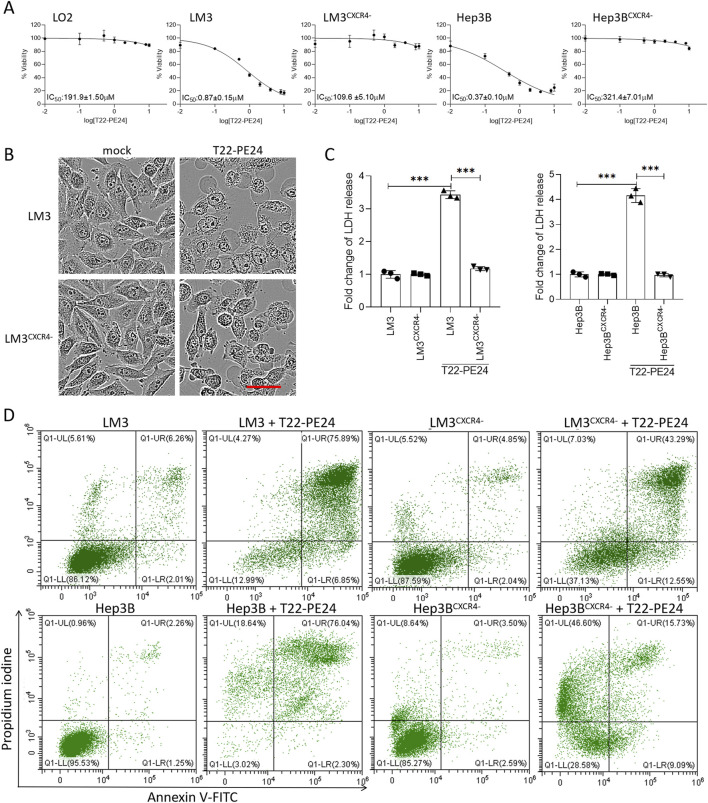
T22-PE24 inhibited the proliferation of HCC cells by activating pyroptosis. **(A)** IC_50_ of LO2, LM3, LM3^CXCR4−^, Hep3B, and Hep3B^CXCR4−^ cells after incubation with T22-PE24. **(B)** Images of cell morphology in LM3 and LM3^CXCR4−^ after incubation with 1 μM T22-PE24 determined by IncuCyte. Scale bar = 50 μm. **(C)** Quantification analysis of the LDH release in LM3, LM3^CXCR4−^, Hep3B, and Hep3B^CXCR4−^ cells after incubation with 1 μM T22-PE24. (Mean ± SD shown. ***p < 0.001 in a two-way ANOVA, followed by Sidak’s multiple-comparison test.) **(D)** Flow cytometric analysis of pyroptosis induced by 1 μM T22-PE24. The upper right quadrant shows the pyroptotic cells.

We evaluated the inhibitory effects on LO2, LM3, LM3^CXCR4−^, Hep3B, and Hep3B^CXCR4−^ after the treatment with T22-PE24 at 24 h (Figure 2). Compared to the LO2 and sgCXCR4 cell lines, T22-PE24 demonstrated strong inhibitory effects on CXCR4-expression LM3 and Hep3B. The IC_50_ value of T22-PE24 was significantly lower in LM3 (0.87 ± 0.15 μM) and Hep3B (0.37 ± 0.10 μM) than in LO2 (191.9 ± 1.50 μM), LM3^CXCR4−^ (109.6 ± 5.10 μM), and Hep3B^CXCR4−^ (321.4 ± 7.01 μM) (Figure 2A). These *in vitro* cytotoxicity results suggested that T22-PE24 selectively accumulated in HCC cells, driven by the targeting capability of the T22 domain.

The antitumor effect of T22-PE24 on HCC cells was evaluated to investigate the mechanism that triggers cell death by observing the cell morphology, detecting LDH release, and analyzing the annexin V/PI level. After the incubation with T22-PE24, LM3 cells exhibited swelling and developed numerous bubble-like protrusions on the surface of the cellular membrane, while characteristics were absent in LM3^CXCR4−^ (Figure 2B). These unique morphological characteristics of LM3 suggested that T22-PE24 triggered pyroptosis rather than apoptosis. Moreover, we detected the release of LDH in LM3, LM3^CXCCR4−^, Hep3B, and Hep3B^CXCR4−^ after the treatment with T22-PE24 at 24 h. The levels of LDH released from LM3 and Hep3B were significantly higher than those from LM3^CXCR4−^ and Hep3B^CXCR4−^ (Figure 2C). The pyroptotic cells were permeable to PI, while the apoptotic cells were not. As shown in Figure 2D, significant increases in the PI fluorescent signals were observed in the treated LM3 and Hep3B cells, and the sgCXCR4 cells showed a fluorescence intensity of PI in comparison with mock cells. Taken together, those data indicated that T22-PE24 would trigger pyroptosis in CXCR4-expression HCC cells.

**FIGURE 3:**
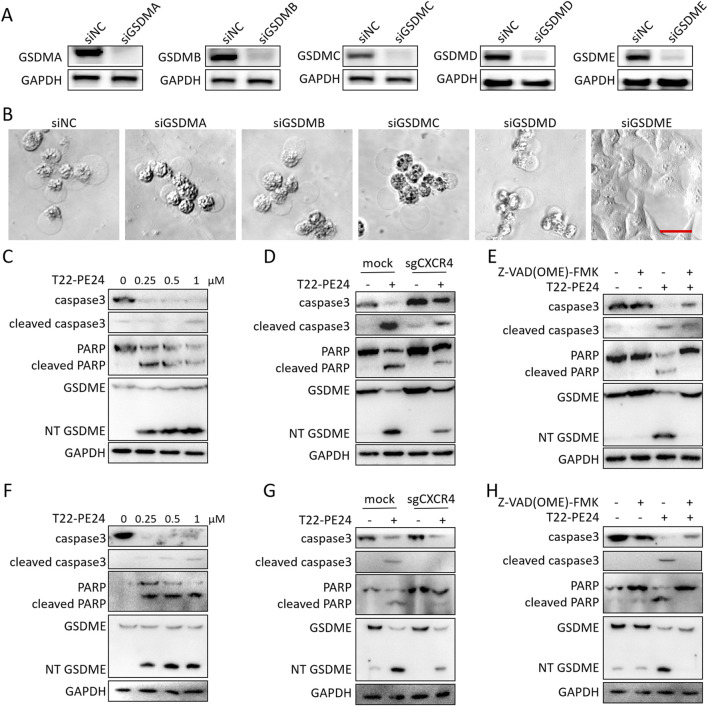
T22-PE24, targeting CXCR4, activated pyroptosis via the caspase3/GSDME pathway. **(A)** Western blot analysis of the silence expression effect in LM3 transfected with siGSDMA, siGSDMB, siGSDMC, siGSDMD, or siGSDME for 48 h. **(B)** Images of cell morphology in LM3 transfected with siGSDMA, siGSDMB, siGSDMC, siGSDMD, or siGSDME after incubation with 1 μM T22-PE24. **(C)** Western blot analysis of LM3 treated with 0 μM, 0.25 μM, 0.5 μM, or 1 μM T22-PE24 for 24 h. **(D)** Western blot analysis of LM3 and LM3^CXCR4−^ cells treated with or without 0.5 μM T22-PE24 for 24 h. **(E)** Western blot analysis of LM3 treated with 0.5 μM T22-PE24 and pretreated with or without 40 μM Z-VAD (OME)-FMK for 24 h. **(F)** Western blot analysis of Hep3B treated with 0 μM, 0.25 μM, 0.5 μM, or 1 μM T22-PE24 for 24 h. **(G)** Western blot analysis of Hep3B and Hep3B^CXCR4−^ cells treated with or without 0.5 μM T22-PE24 for 24 h. **(H)** Western blot analysis of Hep3B treated with 0.5 μM T22-PE24 and pretreated with or without 40 μM Z-VAD (OME)-FMK for 24 h.

We further explored the mechanism by which T22-PE24 triggered pyroptosis. As shown in Figures 3A, B, the knockdown of GSDMA, GSDMB, GSDMC, or GSDMD had no impact on T22-PE24-induced pyroptosis, whereas the knockdown of GSDME effectively prevented this cell death process. Thus, we subsequently investigated whether T22-PE24 induced pyroptosis via the GSDME signaling pathway. Western blot analysis showed that the levels of cleaved caspase-3, PARP, and GSDME increased in a concentration-dependent manner in LM3 and Hep3B cells treated with T22-PE24 (Figures 3C, F). The knockout of CXCR4 in LM3 and Hep3B could rescue the expression level of cleaved caspase-3 and GSDME activated by T22-PE24 (Figures 3D, G). Moreover, caspase inhibitor Z-VAD (OME)-FMK could also inhibit the expression of cleaved caspase-3 and GSDME induced by T22-PE24 (Figures 3E, H). Hence, T22-PE24 activated caspase-3, following activated GSDME. Combined with the result of the release of LDH, T22-PE24 was suggested to lead to pyroptosis of HCC cells.

### Antitumor effect of T22-PE24 in tumor-bearing mice

**FIGURE 4:**
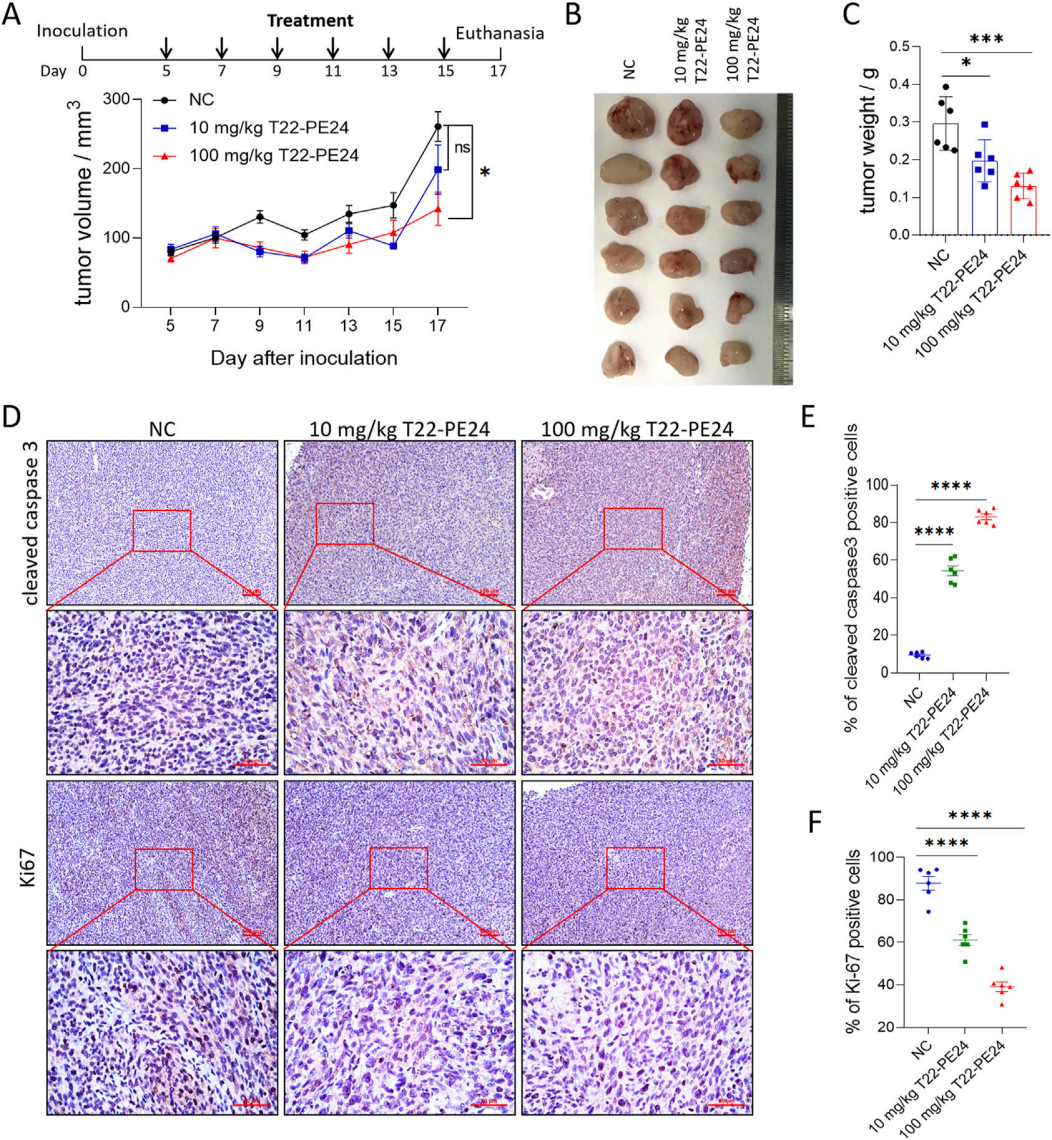
*In vivo* antitumor efficacy of T22-PE24 in tumor-bearing mice. **(A)** Tumor growth was measured over time. T22-PE24 was injected every 2 days for a total of six times. **(B)** Photographs of solid tumors in each treatment group after 17 days. **(C)** Tumor weight of different treatment groups at the end of the experiment. **(D)** IHC images of the tumor sections stained with cleaved caspase-3 or Ki67 after various treatments. **(E)** Quantification analysis of cleaved caspase-3-positive cells. Each dot represented a single tumor. **(F)** Quantification analysis of Ki67-positive cells. Each dot represented a single tumor. Mean ± SEM is shown. ns means no significance. *p < 0.05, ***p < 0.001, ****p < 0.0001. Tumor growth curves used two-way ANOVA followed by Tukey’s multiple-comparison test. Tumor weights, cleaved caspase-3, and Ki67 expression in tumor tissues used a one-way ANOVA, followed by Tukey’s multiple-comparison test.

The tumor inhibitory effect of T22-PE24 on LM3 tumor-bearing mice was evaluated by monitoring tumor growth and other pathophysiological changes. The residual endotoxin of T22-PE24 showed that T22-PE24 used for *in vivo* experiments featured little endotoxin (Supplementary Figure S1H). The tumor size and tumor weight were significantly reduced by treatment with 100 mg/kg of T22-PE24 compared with the physiological saline negative control (NC) (Figures 4A–C). IHC examination of the tumor sections revealed significant apoptosis/pyroptosis and anti-proliferation effects in tumor tissues after treatment with T22-PE24 (Figures 4D–F). This result indicated that T22-PE24 was attributed with a certain antitumor efficiency.

The *in vivo* safety of T22-PE24 was evaluated by monitoring the body weight of the mice and markers of acute nephrotoxicity and hepatotoxicity and histopathological examination of tissues. Compared with the physiological saline negative control group, the body weight did not change significantly in the T22-PE24 treatment groups (Supplementary Figure S2B), indicating that T22-PE24 represents a safe formulation. Pathological histology analysis found that the heart, lung, kidney, liver, and spleen showed no obvious cellular damage induced by T22-PE24 (Supplementary Figure S2C). In addition, compared to the control group, both the 10 mg/kg and 100 mg/kg T22-PE24 groups showed almost no effect on the levels of AST, ALT, Cr, or BUN (Supplementary Figures S2D–G). These results suggested that T22-PE24 does not induce systemic adverse effects in mice.

### Innate immune activation of T22-PE24 in complete-immunity mice

**FIGURE 5:**
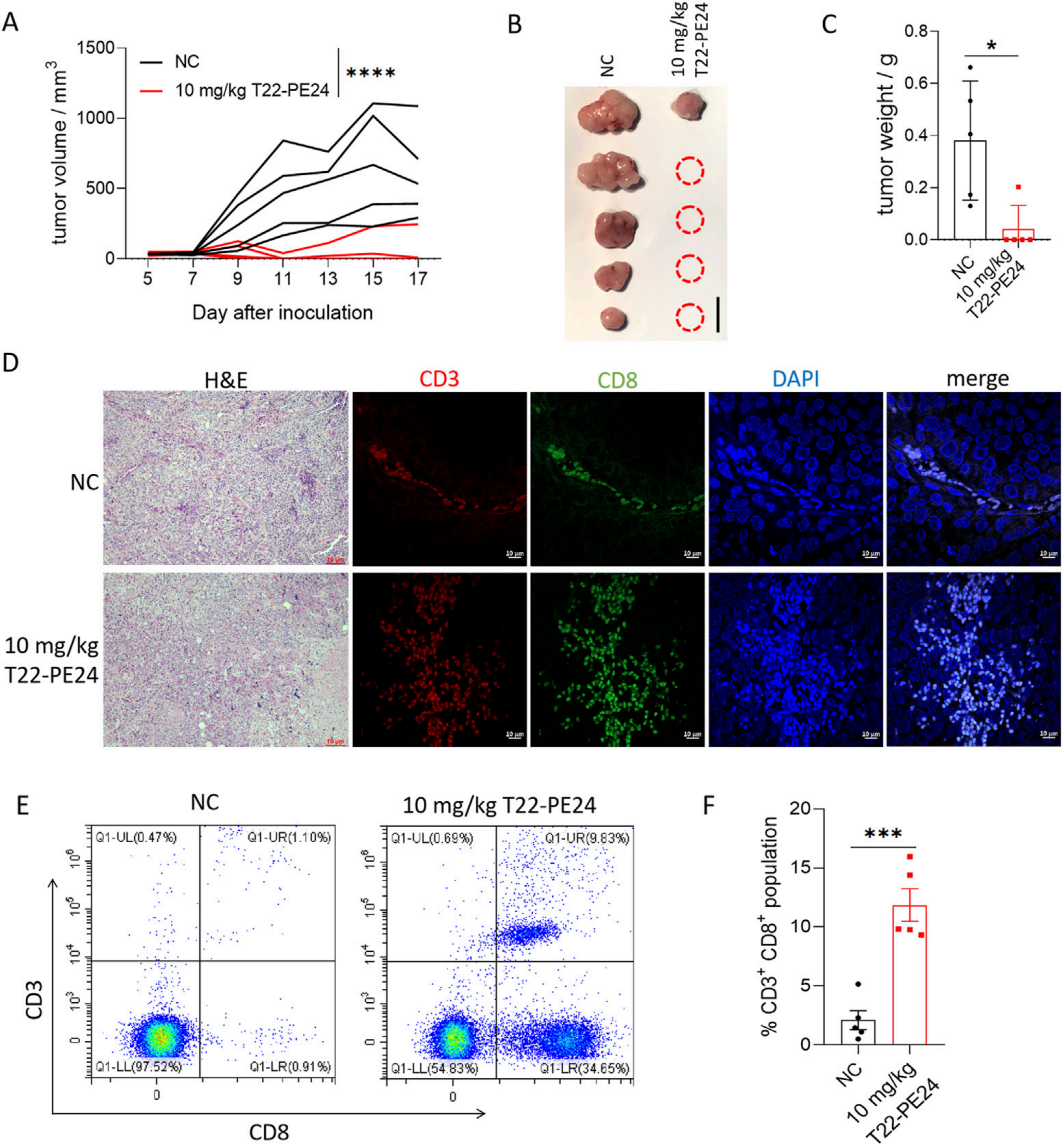
*In vivo* immune activation efficacy of T22-PE24 in tumor-bearing mice. **(A)** Tumor growth was measured over time. T22-PE24 was injected every 2 days for a total of six times. **(B)** Photographs of solid tumors in each treatment group after 17 days. **(C)** Tumor weight of different treatment groups at the end of the experiment. **(D)** H&E and IF images of the tumor sections stained with CD3 and CD8 after various treatments. **(E)** Representative flow cytometry analysis of the CD3^+^ CD8^+^ T cell subgroup in PBMCs. **(F)** Quantification of CD3^+^ CD8^+^ population in PBMCs. Mean ± SEM is shown. *p < 0.05, ***p < 0.001, and ****p < 0.0001. Tumor growth curves used two-way ANOVA, followed by Tukey’s multiple-comparison test. Tumor weights used a one-way ANOVA followed by Tukey’s multiple-comparison test. Quantification of CD3^+^ CD8^+^ population analysis was performed by Student’s t-test.

Pyroptosis activates the innate immune response and reprograms the immune tumor microenvironment. To examine the role of T22-PE24 in immune activation in HCC, we tested the T22-PE24 against HCC cells in complete-immunity C57 mice. Individual tumor growth curves, tumor volumes, and mean tumor weight revealed that low-dose T22-PE24 treatment led to the rejection of four of five tumors (Figures 5A–C). We also performed H&E and IF analysis to confirm the infiltrating CD3^+^ CD8^+^ T cells in tumor tissues. We found that the number of infiltrating CD3^+^ CD8^+^ T cells was significantly increased after T22-PE24 treatment (Figure 5D). By collecting PBMCs, we found that T22-PE24 treatment induced a higher proportion of CD3^+^ CD8^+^ T cells in comparison with the NC group (Figures 5E, F). Collectively, these results suggest that T22-PE24 effectively and globally reprogrammed the immune tumor microenvironment (TME) of HCC by increasing effector immune cells via inducing HCC cell pyroptosis.

## Discussion

This research was carried out to evaluate the potential of the CPPs with tumor-targeting properties to deliver a cytotoxic payload to HCC cells. We explored using a CXCR4-targeting peptide, T22, fused with cytotoxic peptide PE24, as a novel therapeutic strategy for CXCR4^+^ HCC cells. T22 was chosen as the nanocarrier due to its well-established role as a CXCR4 antagonist and its ability to target CXCR4-expressing cells (Masuda et al., 1992). Initially, T22 was designed to inhibit human immunodeficiency virus (HIV) infection (Murakami et al., 1999). Our previous study suggested that T22 exhibited dual functionality by targeting CXCR4 and facilitating cargo release within the cells in melanoma (Zhao et al., 2023). Our data underscored the T22 carrier’s ability to selectively deliver to CXCR4^+^ tumor cells. This selectivity minimizes the risk of systemic toxicity, which is further corroborated by the *in vivo* safety data. These suggested that T22-PE24 could provide a safe and effective approach for targeting CXCR4^+^ tumors.

Multiple studies, including data from TCGA and our own results, have shown that CXCR4 was highly expressed in HCC cells compared to normal cells. As a result, we decided to use T22 fused with a cytotoxic agent, PE24. Observing under TEM and considering the particle size, we found that T22-PE24 self-assembled into nanoparticles. Regarding how T22-PE24 nanoparticles are formed, Sanchez-Garcia et al. (Sanchez-Garcia et al., 2018) reported that short protein segments fused to carrier protein using cationic end-terminal tags could self-assemble into stable nanoparticles that were intrinsically functional with self-delivery properties.

When evaluated for its antitumor mechanism, T22-PE24 exhibited CXCR4 specificity and cytotoxic activity. T22-PE24 nanotoxin activated caspase-3 signaling and followed PARP and GSDME cleaving, which corresponded to apoptosis and pyroptosis, respectively. In combination with IncuCyte and LDH release experiments, we indicated a predominant pyroptosis activation of T22-PE24. In 2020, it was reported that cleaved GSDME activated pyroptosis, further activating the antitumor immune response and inhibiting tumor growth (Zhang et al., 2020). Pyroptosis has gained attention as a potential mechanism for combating tumors due to its pro-inflammatory nature, which may stimulate an immune response against tumor cells. Our data indicated that T22-PE24 enabled targeted pyroptotic cell death in HCC cells with minimal off-target effects.

Falgas et al. (2020b) and Rioja-Blanco et al. (2022b) have extensively studied the *in vivo* biodistribution of T22-GFP. They show that T22-GFP accumulates in CXCR4 overexpressing tumor tissues, with minimal accumulation in other organs. Our *in vivo* study found that none of the treatments caused significant alterations in mouse body weight, organ damage, or alterations in blood biochemistry, further suggesting the excellent biosafety of T22-PE24. Our *in vivo* study further demonstrated that the treatment was less effective in immunodeficient mice than in immunocompetent mice despite the same dosing regimen. Given the minimal off-target effects observed in our *in vivo* toxicity studies, T22-PE24 holds potential as a safer, more effective alternative to conventional chemotherapy, which often lacks such precision and comes with significant adverse effects. In immunodeficient mice, T22-PE24 only affected a small fraction of the tumor-cell population, whereas it targeted almost the entire tumor in immunocompetent mice, highlighting the crucial role of the immune system in enhancing the therapeutic efficacy (Wang et al., 2020). Notably, a markedly increased infiltration of CD3^+^ CD8^+^ T cells was recorded in tumors and PBMCs treated with T22-PE24. Thus, pyroptosis-induced inflammation within the tumor microenvironment can enhance antitumor immunity, suggesting that T22-PE24 may have synergistic potential when combined with other immunomodulatory therapies. However, the immunogenic consequences of T22-PE24-induced pyroptosis remain to be fully elucidated and warrant further exploration in immune-competent models.

## Conclusion

**FIGURE 6:**
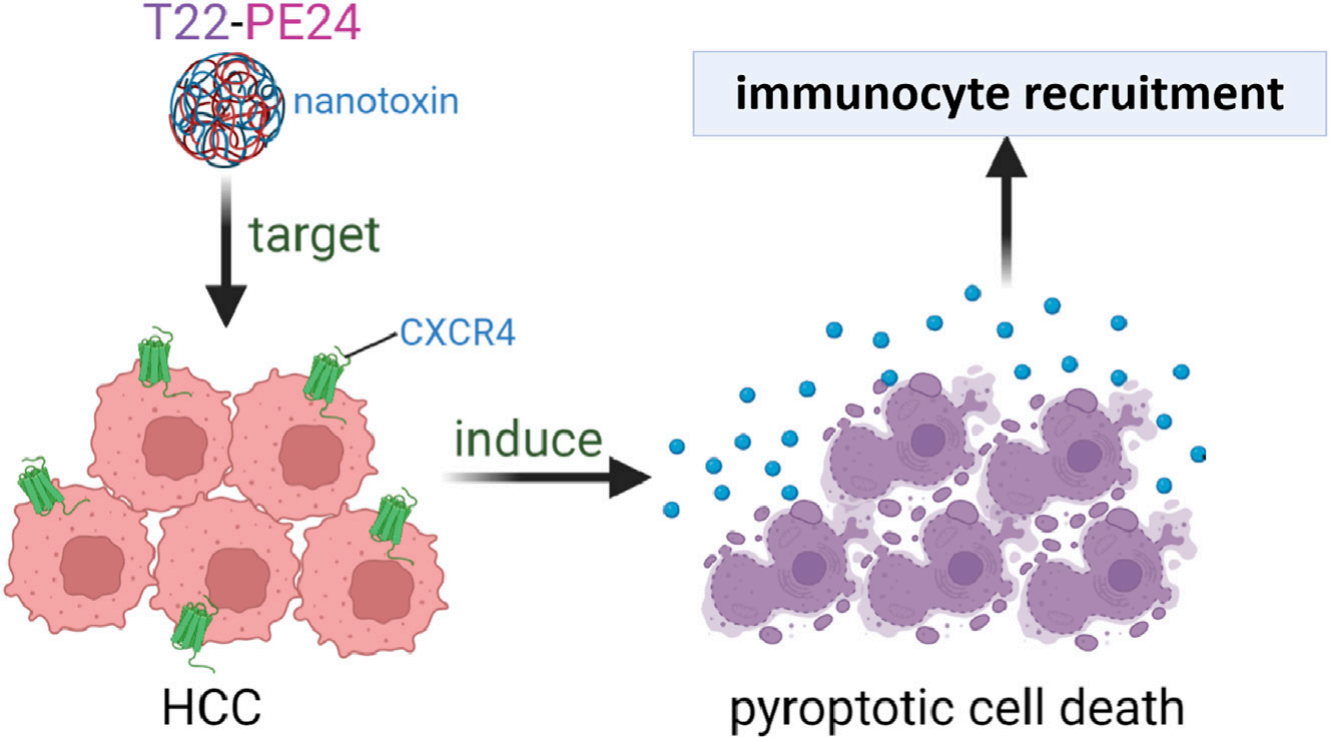
The self-assembling nanotoxin T22-PE24, specifically targeting CXCR4, was designed to selectively deliver the cytotoxic agent PE24 to HCC cells, leading to immunogenic pyroptotic cell death.

In summary, we successfully conjugated PE24 with the CXCR4-targeting CPP T22. T22-PE24 offers a targeted approach to induce pyroptosis, specifically in HCC cells. The fusion protein T22-EGFP was confirmed to be efficiently internalized and accumulated within CXCR4^+^ HCC cells. The particle size and zeta potential of T22-PE24 improved its self-assembling activity. T22-PE24 subsequently resulted in greater HCC cell death by releasing LDH and initiating a cascade of caspase-3/GSDME reactions. T22-PE24 exhibited excellent antitumor efficacy with no adverse effects. Our studies suggest that T22-PE24 holds promise as a targeted therapeutic agent for HCC, offering a potent and specific approach to cancer treatment (Figure 6).

## Data Availability

The datasets presented in this study can be found in online repositories. The names of the repository/repositories and accession number(s) can be found in the article/Supplementary Material.
