# Supplementary figures and images for: Cancer immunogenic cell death via pyroptosis with CXCR4-targeted nanotoxins in hepatocellular carcinoma
Source: Front Bioeng Biotechnol. 2024 Nov 4;12:1433126. doi: 10.3389/fbioe.2024.1433126 (PMC11570815; doi:10.3389/fbioe.2024.1433126)

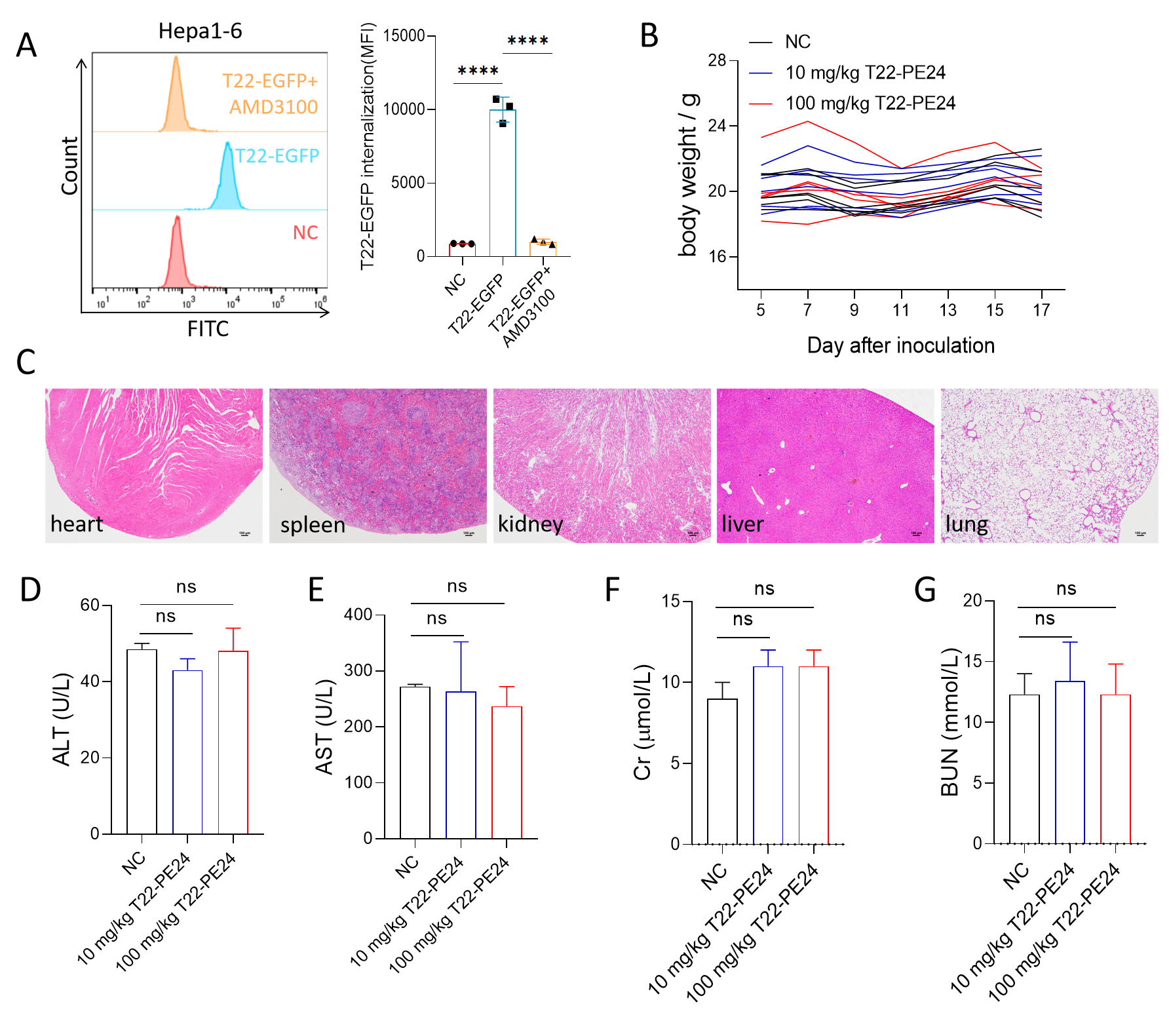

Supplement: Supplementary file 1 [file Image2.TIF]

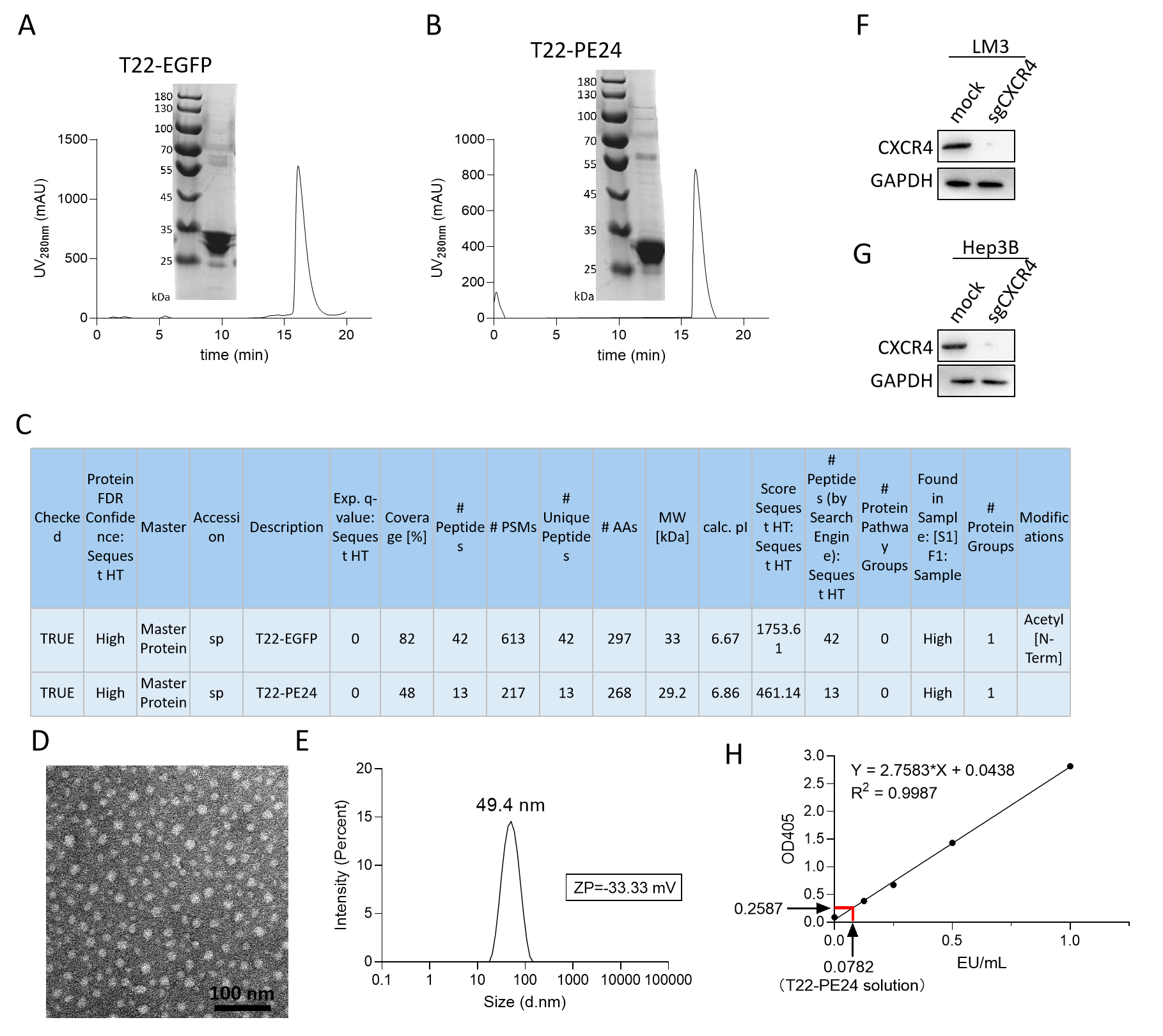

Supplement: Supplementary file 2 [file Image1.TIF]

Figure S1 F-G

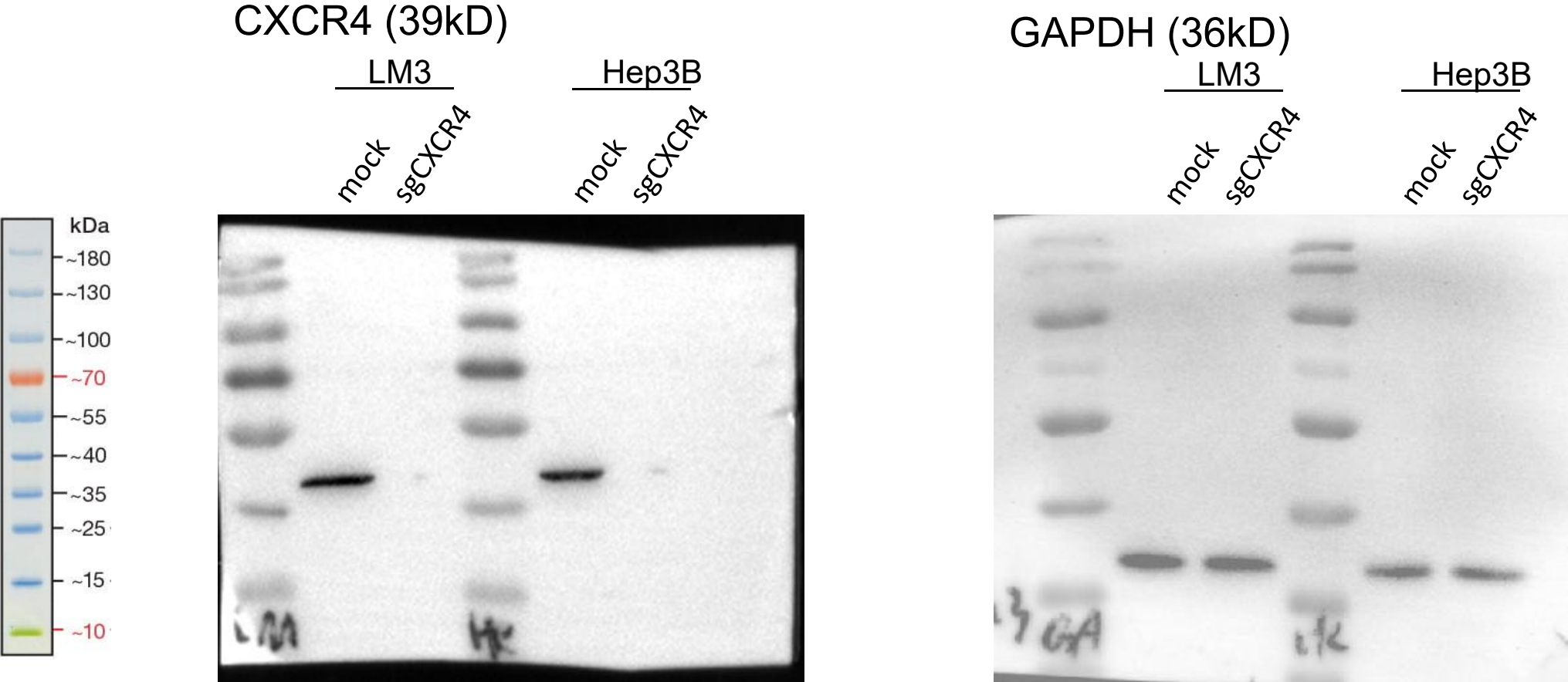

### Figure 3 C-E

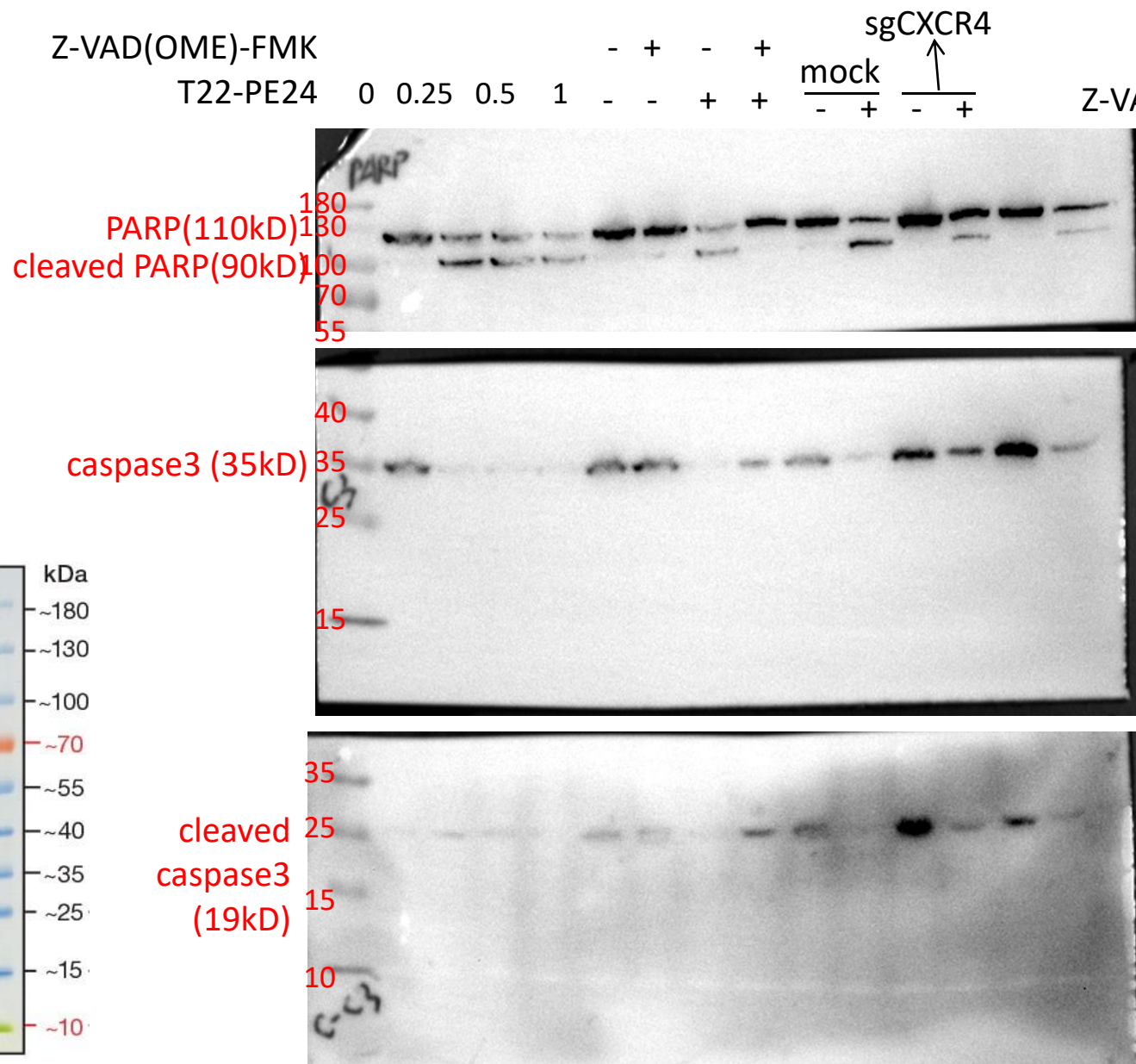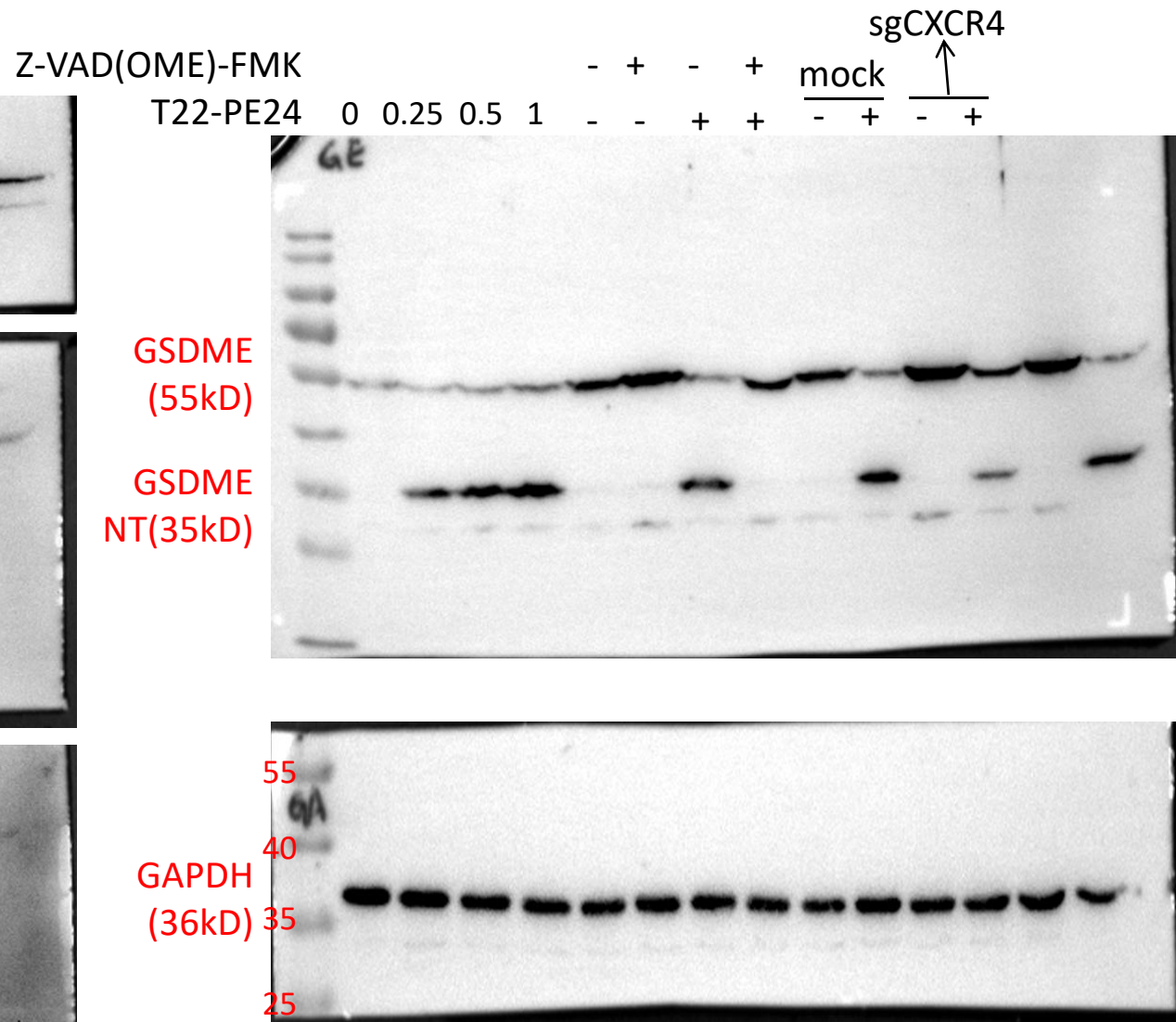

| Z-VAD(OMe)-FMK |                    |   |      |     |   |   |   |   |   | sgCXC4 |   |
|----------------|--------------------|---|------|-----|---|---|---|---|---|--------|---|
| T22-PE24       |                    | 0 | 0.25 | 0.5 | 1 | - | + | - | + | mock   |   |
|                |                    |   |      |     |   | - | - | + | + | -      | + |
| 180            | PARP(110kD)        |   |      |     |   |   |   |   |   |        |   |
| 130            |                    |   |      |     |   |   |   |   |   |        |   |
| 100            | cleaved PARP(90kD) |   |      |     |   |   |   |   |   |        |   |
| 70             |                    |   |      |     |   |   |   |   |   |        |   |
| 55             |                    |   |      |     |   |   |   |   |   |        |   |

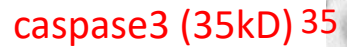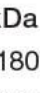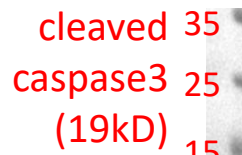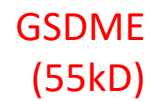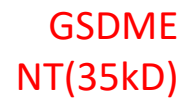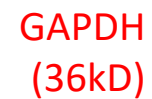

Supplement: Supplementary file 3 [file DataSheet1.pdf]
